# Reliability of prisoners’ survey responses: comparison of self-reported health and biomedical data from an australian prisoner cohort

**DOI:** 10.1186/s12889-021-12460-7

**Published:** 2022-01-10

**Authors:** Tony Gerard Butler, Mathew Gullotta, David Greenberg

**Affiliations:** 1 School of Population Health, University of New South Wales, 2052 Sydney, NSW Australia; 2 The Wellbeing Group, 2040 Sydney, NSW Australia; 3 Statewide Community Court Liaison Service, Justice Health and Forensic Mental Health Network, 2036 Sydney, NSW Australia; 4 School of Psychiatry, University of New South Wales, 2052 Sydney, NSW Australia

**Keywords:** Self-reported, Health surveys, Biomedical, Objective tests, Reliability, Blood borne viruses, Hepatitis, Sexual transmissible infections

## Abstract

**Objective:**

Prisoner health surveys primarily rely on self-report data. However, it is unclear whether prisoners are reliable health survey respondents. This paper aimed to determine the level of agreement between self-report and biomedical tests for a number of chronic health conditions.

**Method:**

This study was a secondary analysis of existing data from three waves (1996, 2001, 2009) of the New South Wales (NSW) Inmate Health Survey. The health surveys were cross-sectional in nature and included a stratified random sample of men (*n*=2,114) from all NSW prisons. Self-reported histories of hepatitis, sexually transmissible infections, and diabetes were compared to objective biomedical measures of these conditions.

**Results:**

Overall, the sensitivity (i.e., the respondents who self-reported having the condition also had markers indicative of the condition using biomedical tests) was high for hepatitis C (96%) and hepatitis B (83%), but low for all other assessed conditions (ranging from 9.1% for syphilis using RPR to 64% for diabetes). However, Kappa scores indicated substantial agreement only for hepatitis C. That is, there were false positives and false negatives which occurred outside of chance leading to poor agreement for all other assessed conditions.

**Conclusions:**

Prisoners may have been exposed to serious health conditions while failing to report a history of infection. It may be possible that prisoners do not get tested given the asymptomatic presentation of some conditions, were unaware of their health status, have limited health-service usage preventing the opportunity for detection, or are subject to forgetting or misunderstanding prior test results. These findings demonstrate the importance of the custodial environment in screening for health conditions and referral for treatment should this be needed. Testing on entry, periodically during incarceration, and prior to release is recommended.

## Introduction

The reliability of self-reported health information is important as it forms the basis of feedback to researchers who rely on it to determine individual or population health needs. Comparing self-report to objective biological markers is one method of checking data accuracy but may differ due to: forgetting or misunderstanding prior test results; willingness to report certain condiitons; lack of awareness of a condition, particularly if asymptomatic; or inaccurate or incomplete testing. Several studies have examined the reliability of self-report among different populations. Community-based respondents have been found to provide health information that is generally consistent with medical tests or historical records for a variety of health outcomes [1–4]. For example, a community sample of Chinese respondents found moderate to good agreement between self-report and tests of diabetes and hypertension [5]. A European study found good agreement between self-report of fractures and clinical records, with only 11% reporting a fracture without confirmatory clinical records (false positives) and 7% reporting no fracture when records indicated otherwise (false negative) [6]. Other populations self-report of their health status is shown to be less consistent with the results of biomedical tests. This may reflect poor accuracy in self-reporting or a lack of testing in those who may be asymptomatic. Nevertheless, the degree of agreement between self-reported hepatitis A, hepatitis B, and hepatitis C status and serological testing among intravenous drug users [7, 8] and men who have sex with men [9] has been found to be poor. A review found that 46-95% of intravenous drug users tested positive for exposure to hepatitis B but reported no history of infection [10].

Relatively few studies have examined the reliability of self-reports of prisoners’ health status. An Australian study found that prisoners’ self-report of traumatic brain injury to be highly accurate as 83% of those that self-reported a traumatic brain injury had concordant hospital records of brain injury [11]. The reliability of self-report of other conditions among prisoners such as hepatitis, sexually transmissible infections, and non-infectious conditions are less well researched. Recently, Bai and colleagues [12] examined agreement between self-reported chronic health conditions and medical records of prisoners in the United States. The authors found that the level of agreement between self-report and medical records varied by sex, with better concordance for females than males, and type of condition. Overall, prisoners in this sample were poor at self-reporting renal/kidney disease (κ = 0.50), hypertension (κ = 0.57), and hepatitis C (κ = 0.66) but more accurate for HIV and diabetes (κs =0.89) [12].

Prisoners represent an important group in terms of public health and healthcare as this population, in Australia and elsewhere, are found to be at an increased risk of exposure to blood borne viruses [13] and sexually transmissible infections [14, 15] and cycle between prison and the community. However, the prevalence and control of non-infectious disease such as diabetes is less well described among the prisoner population, with existing studies finding conflicting results [16, 17]. As such, there are important implications for improving healthcare in prisons, the future use of health services for those released from custody, and identifying and minimizing the risk of deterioration from uncontrolled non-infectious health conditions and the transmission of infectious diseases to others. This paper examines the validity of self-reported chronic health conditions in a sample of men imprisoned in an Australian jurisdiction (New South Wales). Data from several cross-sectional Australian inmate health surveys were used to compare the level of agreement between self-reported histories of hepatitis, sexually transmissible infections, and diabetes with objective biomedical measures.

## Method

This article presents a secondary analysis of data from three waves of prisoner health surveys conducted in an Australian jurisdiction. The 1996 [18], 2001 [19], and 2009 [20] New South Wales (NSW) Inmate Health Surveys represent cross-sectional surveys that provided comprehensive data on the physical health, mental health, and health risk behaviours of age, sex, and Aboriginal stratified random samples of men and women prisoners from all adult correctional centres in NSW. Although data for both men and women were collected in the original surveys, this study only examined the data from men. Men represent over 90% of the total prisoner population in NSW [21].

The methods for the health surveys are described in detail elsewhere [18–20].Participants were included if they were over 18 years of age, spoke sufficient English to be interviewed, and were able to provide informed consent. Health conditions examined in this study were diabetes, hepatitis B, hepatitis C, chlamydia, herpes simplex virus type-2, and syphilis. Prisoners participated in face-to-face interviews (1996, 2001, and 2009) and telephone interviews (2009) conducted by registered nurses trained on the study protocol to collect self-reported information regarding their physical health, mental health, and health risk behaviours. Participants were asked if a doctor had ever diagnosed any of a number of specified health conditions or illnesses (0=*no*, 1=*yes*). Prisoners also underwent testing for blood-borne viral infections and sexually transmissible infections using blood and urine samples. Non-fasting blood sugar (random blood glucose) tests were conducted and levels were classified into *unlikely* (<5.5 mmol/L), *possibly* (>=5.5 mmol/L to 11 mmol/L), and *likely* (>11 mmol/L) that diabetes may be present based on this test. Random blood glucose levels between 5.5mmol/L to 11mmol/L may require further testing for diabetes. Hepatitis B core antibody (HBcAb) and hepatitis C antibody (HCAb) were tested to determine exposure to either of these infections. Chlaymdia was screened for by chlamydia polymerase chain reaction (PCR), herpes simplex virus type-2 (HSV-2) was identified by the concentration of HSV-2 specific antibodies, and reactive rapid plasma regain (RPR) and reactive treponema pallidium particle agglutination (TPPA) were used to test for syphilis. Positive RPR and TPPA were confirmed using fluorescent treponemal antibody test.

### Ethics

Ethics approval and informed consent to participate in the health surveys is described in the original publications [18–20]. Approval for the use of the data to examine the health of men of specific offender groups (e.g., sex offenders) was provided by the Justice Health and Forensic Mental Health Network Human Research Ethics Committee (G70/14).

### Statistical analysis

To assess the level of agreement between the self-reported data and biomedical measures, sensitivity, specificity and κ coefficients were each calculated. Biomedical measures were treated as the reference or the “gold standard” for diagnosis. Sensitivity refers to the true positive rate which was calculated as the percentage of respondents who were diagnosed with the condition among those who self-reported having the condition. Specificity refers to the true negative rate which was calculated as the percentage of individuals who self-reported not having the condition among those who were found to not have the concordant test results. The κ coefficient is a more robust measure than simple percent agreement and also takes into account the agreement occurring by chance. The κ coefficient was divided into four categories: 0-0.40 “poor-to-fair agreement”; 0.41 to 0.60 “moderate agreement”; 0.61-0.80 “substantial agreement”; and 0.81-1.0 “excellent agreement” [12]. Statistical analyses were conducted using IBM® Statistical Package for the Social Sciences, version 23.

## Results

A total of 2,114 men participated in one of the three prisoner health surveys. The average age of the sample was 33.74 years (*SD=*12.25 years). A total of 664 participants identified as Aboriginal or Torres Strait Islander (30.3% of the sample). There were incomplete or unavailable (“missing”) data for the self-report by biomedical tests from a number of participants (Table 1). Sensitivity, specificity and κ coefficients were calculated on the available valid data.

**Table 1:** Self-reported health conditions by serological status for hepatitis and sexually transmissible infections

			Condition test results				*X* ^2^, *p* (Φ/ Φ_C_)	Sensitivity	Specificity	κ, *p*
Self-report			Blood sugar levels							
Diabetes		Total	Likely (>11 mmol/L)	Possible (≥5.5 mmol/L to 11 mmol/L)		Unlikely (<5.5 mmol/L)				
Yes		36 (1.7%)	7	16		13	X^2^=45.723, *p*<.001 (0.191)	63.9%	98.3%	κ = 0.022, p=.047
No		1,222 (57.8%)	25	452		745				
Missing		856 (40.5%)								
Hepatitis B			Positive HBcAb		Negative HBcAb					
Yes		121 (5.7%)	100		21		*X* ^2^=186.136, *p*<.001 (0.401)	82.6%	97.4%	κ = 0.332, *p*<.001
No		1,038 (49.1%)	239		799					
Missing		955 (45.2%)								
Hepatitis C			Positive HCAb		Negative HCAb					
Yes		415 (19.6%)	398		17		*X* ^2^=787.788, *p*<.001 (0.782)	95.9%	97.8%	κ = 0.770, *p*<.001
No		874 (41.3%)	121		753					
Missing		825 (39.0%)								
Chlamydia			Positive		Negative					
Yes		46 (2.2%)	0		46		*X* ^2^=1.060, *p*=.303 (0.030)	0%	95.9%	κ= -0.029, *p*=.303
No		1,109 (52.4%)	25		1,084					
Missing		959 (45.4%)								
Herpes (genital)			Positive		Negative					
Yes		25 (1.2%)	13		12		*X* ^2^=29.069, *p*<.001 (0.182)	52.0%	98.4%	κ = 0.126, *p*<.001
No		845 (40.0%)	114		731					
Missing		1,241 (58.7%)								
Syphilis			Reactive RPR		Non-reactive RPR					
Yes		44 (2.1%)	4		40		*X* ^2^=14.818, *p*<.001 (0.162)	9.1%	92.8%	κ = 0.123, *p*<.001
No		524 (24.8%)	6		518					
Missing		1,546 (73.1%)								
			Reactive TPPA		Non-reactive TPPA					
Yes		42 (2.0%)	12		30		*X* ^2^=42.733, *p*<.001 (0.306)	28.6%	93.0%	κ = 0.297, *p*<.001
No		415 (19.6%)	15		400					
Missing		1,657 (78.4%)								

The information in this table is based on the self-report and biomedical data. The sample size in this table reflect the number of participants who were able to be coded on each item. Some responses could not be coded because of the amount and quality of the information

Biomedical testing revealed that hepatitis C was the most common condition among the sample with 419 men (33.8%) that tested positive, while chlamydia was the least common with only 25 men (2.2%) having tested positive.

Using biomedical data as the gold standard measure for the medical condition, sensitivity was moderate for hepatitis B and high for hepatitis C, but low for sexually transmissible infections ranging from 9.1% (for RPR) for syphilis to 52.0% for herpes simplex type-2 (genital). There were 46 men who reported having chlamydia but had a negative test result, which may be indicative of cleared or treated infection. However, 25 men who did not report chlamydia tested positive. Specificity was high for all the variables ranging from 92.8% for syphilis (using RPR marker) to 98.4% for herpes. κ coefficients showed substantial agreement for hepatitis C and poor to fair agreement for all other variables.

## Discussion

We compared self-reported diabetes, hepatitis B and hepatitis C, and sexually transmissible infections to results from objective biomedical tests obtained at the time of the interview for three waves of a large cross-sectional survey of prison inmates. Biochemical validation of self-reported diabetes and exposure to blood-borne viruses (e.g. hepatitis) and sexually transmissible infections provides an accurate means for assessing the reliability of self-report among prisoners.

Many have suggested that self-reports of hepatitis B and hepatitis C are unreliable and lack sufficient validity to be considered useful [10, 22]. This may be due to the potentially asymptomatic nature of these infections and therefore unawareness of being infected. However, we found a high level of agreement between self-report and serological testing for hepatitis C antibodies, with high sensitivity (96% who self-reported the condition tested positive) and specificity (98% who denied the condition tested negative). The κ coefficients appeared to suggest a substantial level of agreement between self-report and biomedical tests for hepatitis C.

The sensitivity and level of agreement between the self-report and biomedical tests were not as high for hepatitis B (86% who self-reported the condition tested positive) and were even poorer for the sexually transmissible infections included in this analysis. A significant proportion of those who denied exposure tested positive for these conditions. Overall, these findings are similar to that of previous studies that showed prisoners [12] and other groups (e.g., intravenous drug users) have increased exposure to certain infectious diseases [7, 9].

The notion that prisoners may have exposure to serious health conditions while failing to report a history of infection potentially has implications for transmission of infections such as hepatitis B and hepatitis C. It may be possible that prisoners do not get tested given the asymptomatic presentation of some of these conditions. Alternatively, it may be reflective of the limited health-service usage among offenders [20] which prevents the opportunity for detection. It is possible that certain conditions such as hepatitis C or STIs have stigma attached to them and so there is a reluctance to get tested. Nevertheless, inaccurately assuming negative blood-borne virus and sexually transmissible infections status may have serious public health consequences [22]. Most prisoners are released from custody and return to the community which may put others at risk if their conditions remain unbeknownst to them and untreated. To this end, these findings reinforce the importance of the custodial environment offering a valuable screening opportunity for these conditions and access to healthcare in prisons. This screening should occur on entry to prison, periodically during incarceration, and prior to release.

With regard to diabetes, the current findings suggested that the specificity and level of agreement between participant’s self-reported history of diabetes and non-fasting blood sugar levels was poor. A total of 25 people self-reported no history of diabetes whilst returning a blood sugar level of above 11.0mmol/L, which is indicative of diabetes as per the current Australian guidelines [23]. However, non-fasting blood glucose levels of 5.5mmol/L – 11.0mmol/L is not a definitive indicator of diabetes, and these results should be interpreted with caution. Unfortunately, non-fasting blood sugar levels could not be examined by the surveys due to limitations in the testing environment. Glycated haemoglobin (HbA1c) tests were not conducted. While contrasting previous findings of moderate to good agreement between self-report and biomedical tests of diabetes among community members, the current results should acknowledge that some people entering the custodial environment may not have previously been informed of their diabetic status. Assessment of diabetes is important given the increased risk for other chronic conditions such as cardiovascular and kidney disease, as well as infection [23]. Diabetes treatment may require lifestyle changes (e.g., diet, cessation and reduction of alcohol and tobacco, physical activity) or medical management which could be initiated within the custodial environment.

Overall, it appears that the self-report of men in custody regarding their health status may be valid for some health conditions (diabetes, hepatitis B and hepatitis C), but not others (such as sexually transmissible infections). Future research may wish to examine whether women in contact with the criminal justice system are more, equally, or less valid health respondents. The sole use of self-report may be result in false-negatives (reporting no history of a condition, with biomedical data suggesting a history) or false-positives (reporting a history of a condition, without confirmatory biomedical data). A combination of both self-report and biomedical data may be useful when attempting to identify the prevalence of chronic conditions among prisoners. Future research should attempt to identify key characteristics that differentiate prisoners who accurately identify their health status to those who do not. Identifying those at risk of serious health conditions who fail to report a history of infection can lead to improvements in public and prison healthcare, as well as screening and treatment within the custodial environment.

## Data Availability

The data that support the findings of this study are available from the Justice Health and Forensic Mental Health Network but restrictions apply to the availability of these data, which were used under license for the current study, and so are not publicly available. Data are however available from the Justice Health and Forensic Mental Health Network upon reasonable request. *Address*: Research and Evaluation Service, Justice Health & Forensic Mental Health Network. 1300 Anzac Parade Malabar NSW 2036; Phone: +61,297,003,833; Email: RED@justicehealth.nsw.gov.au.
